# Effects of Omega-3 Long Chain Polyunsaturated Fatty Acid Supplementation on Cardiovascular Mortality: The Importance of the Dose of DHA

**DOI:** 10.3390/nu9121305

**Published:** 2017-11-30

**Authors:** Barbara J. Meyer, Renate H. M. de Groot

**Affiliations:** 1 School of Medicine, Lipid Research Centre, Illawarra Health & Medical Research Institute, University of Wollongong, Wollongong, NSW 2522, Australia; 2 Welten Institute—Research Centre for Learning, Teaching, and Technology, Open University of The Netherlands, 6419 AT Heerlen, The Netherlands; renate.degroot@ou.nl; 3 Department of Complex Genetics, School for Nutrition, Toxicology and Metabolism, Maastricht University, 6200 MD Maastricht, The Netherlands

**Keywords:** omega-3, long chain polyunsaturated fatty acids, cardiovascular disease, randomised controlled trials, dose of omega-3, omega-3 status

## Abstract

Recent evidence on the relationship between omega-3 long chain polyunsaturated fatty acid (*n*-3 LCPUFA) supplementation and cardiovascular health suggests that *n*-3 LCPUFA may no longer be efficacious. This review summarises the randomised controlled trials (RCTs) that assess the effect of *n*-3 LCPUFA supplementation on cardiovascular mortality. It appears that in the RCTs that showed no effect of *n*-3 LCPUFA on cardiovascular mortality, the dose of *n*-3 LCPUFA (in particular docosahexaenoic acid (DHA)) and hence the *n*-3 LCPUFA status, may not have been sufficiently high to demonstrate the efficacy, and/or the baseline *n*-3 LCPUFA status was already too high. The intention-to-treat analysis (ITT) is the gold standard for analysing RCTs and ITT is used for drug intervention trials where exposure to the drug versus no drug exposure provides two clearly distinct groups to determine the efficacy of the drug being studied. This differs in nutrition trials as often the nutrient of interest being studied is already being consumed by both groups (placebo and active) and therefore a true placebo group with absolutely no intake of the nutrient being studied is highly unlikely. Therefore, in *n*-3 LCPUFA supplementation trials, as there is no clear distinction between the two groups (placebo and *n*-3 LCPUFA), a per-protocol analysis (comparison of groups that includes only those participants that fully completed the original intervention allocation) should be conducted in addition to ITT analysis. Furthermore, blood analysis pre- and post-supplementation should be conducted to ensure that: (1) that the baseline *n*-3 status is not too high, in order to alleviate a potential ceiling effect; and (2) that the dose is high enough and hence the increase in omega-3 status will be high enough in order to assess the efficacy of *n*-3 LCPUFA supplementation.

## 1. Introduction

The health benefits of omega-3 long-chain polyunsaturated fatty acids (*n*-3 LCPUFAs) are numerous and include neurological development [1,2,3], improved cognition [4,5,6], and reduced cognitive decline [7] as well as cardiovascular disease (CVD) benefits [8].

The interest in *n*-3 LCPUFA originates from the documented evidence that Eskimos’ rates of CVD were much lower than those of the Danish population, despite the Eskimos’ high consumption of fat [9]. The high fat intake by the Eskimos was due to high consumption of whale meat and whale fat which contained high amounts *n*-3 LCPUFA [9]. The landmark study by Bang et al. demonstrated the blood-thinning effects of *n*-3 LCPUFA [10]. Since then there have been numerous publications on the cardiovascular health benefits of *n*-3 LCPUFA consumption, but recent meta-analyses suggest otherwise.

However, from observational studies, the rates of cardiac death in the Japanese population per 1000 person years are very low at 2.5, as compared to 17 cardiac deaths per 1000 person years in the Italian population [11]. Traditionally, the Japanese consume on average one fish meal per day which provides approximately 900 mg *n*-3 LCPUFA [12]. The low rates of CVD in the Japanese population have been well documented despite high rates of hypertension and smoking [13]. When the Japanese move to Hawaii and adopt a diet much lower in *n*-3 LCPUFA, risk factors for CVD increase, suggesting that the Japanese protective effect is not purely due to genetics [14]. Furthermore, total *n*-3 LCPUFA intakes of 750 mg per day showed the greatest protection in the age-adjusted mortality (from CVD, coronary heart disease and stroke) in a cross-sectional study of 38 countries [13].

The *n*-3 LCPUFAs are comprised of eicosapentaenoic acid (EPA), docosapentaenoic acid (DPA), and docosahexaenoic acid (DHA). The majority of research has been conducted on EPA and DHA. The cardioprotective effects of *n*-3 LCPUFA include plasma triacylglycerol-lowering effects, anti-inflammatory actions through the production of eicosanoids, generation of proresolving lipid mediators, modulation of cardiac ion channels, and influence on membrane fluidity and downstream cell signalling pathways, as well as anti-thrombotic and anti-arrhythmic effects [15,16,17]. Certainly in animal models, where the underlying mechanisms can be elucidated, DHA reduced the incidence and severity of ventricular arrhythmias in rats, whereas EPA alone had no effect [18]. The physiological mechanisms by which dietary *n*-3 LCPUFAs exert their cardioprotection are explained in detail in a review by McLennan (2014) [19]. It is conceivable that DHA, not EPA, protects us from cardiac arrhythmia and sudden cardiac death. Certainly the GISSI-Prevenzione trial was the first randomised controlled trial (RCT) that demonstrated that 840 mg *n*-3 LCPUFA per day (including 560 mg DHA) resulted in a 20% reduction in total deaths, a 30% reduction in CVD deaths, a 35% reduction in coronary death, and a 45% reduction in sudden cardiac death [8].

However, recently published systematic reviews of *n*-3 RCTs on cardiac mortality have discounted *n*-3 LCPUFA in being efficacious in reducing death from CVD [20,21,22]. There could be a number of reasons for the lack of efficacy of the more recent trials mentioned in these reviews, including low doses used and the lack of control for background omega-3 status. Therefore, the aim of this review was to critique the research manuscripts from these reviews [20,21,22] and to investigate what potential role dose and background fatty acid status played in showing efficacy (or lack thereof) of *n*-3 LCPUFA supplementation with respect to CVD mortality.

## 2. Materials and Methods

The systematic reviews, meta-analyses [20,21], and other review publications [22] that reported the lack of *n*-3 LCPUFA supplementation on CVD mortality were used to obtain the original manuscripts from their reference list. The eligibility criteria for inclusion in this review were research manuscripts that assessed the effect of *n*-3 LCPUFA supplementation on CVD mortality (including coronary heart disease (CHD), stroke, heart failure, and sudden cardiac death). Research manuscripts that did not include CVD mortality as an outcome measure were excluded from this review. The eligible manuscripts were read and the dose, duration, study population, medication use, diet, blood assessments, outcomes measures, and our comments were recorded (Supplemental Table S1).

## 3. Results and Discussion

### 3.1. Randomised Controlled Trials and Dose of DHA

A review of RCTs investigating the effects of supplementation with *n*-3 LCPUFA on cardiovascular health [23] highlights that some RCTs do not support the efficacy of *n*-3 LCPUFA supplementation on cardiovascular mortality. This lack of efficacy could be due to the increased level of medication use by people with heart disease. For example, in the GISSI-P trial less than 50% of people were taking angiotensin-converting-enzyme (ACE) inhibitors, beta-blockers, and cholesterol-lowering medication, whilst in the later trials over 80–90% of people were taking these drugs (Supplemental Table S1). These trials also ranged widely in the sample size, from *n* = 43 (pilot study) to a very large study with 18,645 individuals (Supplemental Table S1). Our Supplemental Table S1 contains RCTs on *n*-3 LCPUFA supplementation and cardiovascular mortality (including CHD, stroke, heart failure, and sudden cardiac death). This review contains a brief chronological description of these studies that assessed cardiovascular mortality and also highlights that the dose of *n*-3 LCPUFA, in particular docosahexaenoic acid (DHA) may not have been sufficient in those RCTs that did not show the efficacy of *n*-3 LCPUFA supplementation. In addition, it shows that the background fatty acid status plays an important role in showing efficacy.

The first randomised controlled trial to publish the efficacy of *n*-3 LCPUFA supplementation (840 mg per day; 280 mg EPA and 560 mg DHA daily—EPA:DHA ratio 1:2) was the GISSI-Prevenzione trial [8]. This trial was a secondary prevention trial in Italian men who had suffered a prior myocardial infarction. The primary outcome measures included a 15% reduction in death and 20% reduction in cardiovascular death. Secondary outcomes included a 20% reduction in all fatal events; 30% reduction in cardiovascular death; 35% reduction in cardiac and coronary deaths; and a 40% reduction in sudden deaths.

The OPACH study (1680 mg per day; 930 mg EPA and 750 mg DHA) was conducted in people on chronic haemodialysis [24] which resulted in 70% reduction in myocardial infarction and a 60% reduction in major coronary events.

The JELIS trial (1800 mg EPA per day) [25] was conducted in a Japanese population where the cardiac death rate per 1000 persons of 2.5 is much lower than the rate of 17 deaths per 1000 individuals in the GISSI trial [8]. Even so, the EPA-rich trial showed a significant 19% reduction in any major coronary event, a 24% reduction in unstable angina, and a 19% reduction in non-fatal coronary events. Even though the omega-3 supplementation was only with EPA, this was done on a background of high dietary intake of DHA of 550 mg per day [14].

The GISSI heart failure trial (280 mg EPA per day and 560 mg DHA per day) [26] resulted in a 9% reduction in time to death, an 8% reduction in actual events, and a 10% reduction in hospital admissions. There was also a secondary outcome measure of a 7% reduction in sudden cardiac death.

The Alpha Omega Trial [27] and the Omega trial [28] were underpowered due to limited events rates. Another underpowered trial [29] with only *n* = 140 per group but using a reasonable dose of omega-3 (1176 mg EPA and 840 mg DHA) resulted in a trend towards significant reduction in all-cause mortality, with event rates of 5% versus 8.5% (*p* = 0.063).

The other remaining studies listed in the Supplemental Table S1 [30,31,32,33,34,35] all involved supplementation with 375 mg DHA per day or less and these studies did not demonstrate significant effects of omega-3 supplementation on CVD outcomes. Interestingly, the Origin left ventricular ejection fraction (LVEF) [31] study used two doses of *n*-3 LCPUFA; namely a 1 g dose (providing 375 mg DHA per day) and a higher 4 g dose (providing 1500 mg DHA per day). The higher 4 g dose (1500 mg DHA per day) did show significant outcomes in left ventricular ejection fraction, flow mediated dilatation, and interleukin-6, whereas the 1 g dose (providing 375 mg DHA per day) was ineffective [31], suggesting that the dose of supplementation, particularly DHA, is important.

Even though some trials were very large, some trials did not have high enough event rates to draw any meaningful conclusions [30,32,35]. Other trials were clearly underpowered [27,28,29,35]. Furthermore, low-dose DHA studies such as the OPERA trial [33] showed that the DHA dose increased from 3.15% to 3.84%, and clearly this change in DHA levels is not large enough to see a significant benefit.

**Figure 1:**
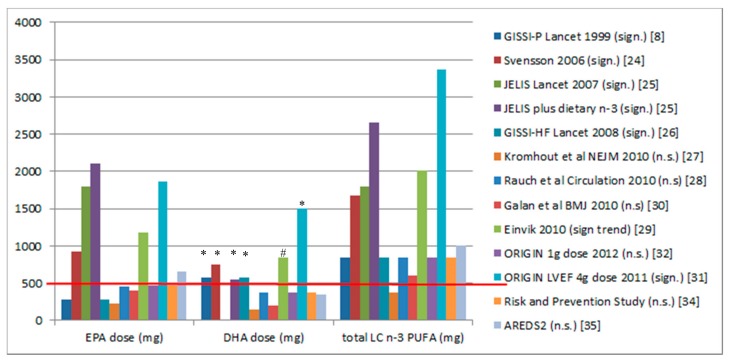
Doses of eicosapentaenoic acid (EPA), docosahexaenoic acid (DHA), and total omega-3 long-chain polyunsaturated fatty acid (*n*-3 LCPUFA) from randomised controlled trials on *n*-3 LCPUFA supplementation and clinical cardiovascular disease (CVD) outcomes. The red line shows that a DHA dose of 500 mg or greater results in significant CVD outcomes. * Significant effects (*p* < 0.05); ^#^ only 140 per group showing a trend towards a significant effect (*p* = 0.063). Sign means significant and n.s. means not significant.

Figure 1 shows the dose of supplementation from the studies listed in Supplemental Table S1. It appears that there is a dose threshold of approximately 500 mg DHA and every study with an at least 500 mg dose of DHA showed CVD benefit. The Japanese consume 550 mg DHA per day, and their rates of cardiac death per 1000 person years are 6.8-fold lower than in the Italian population [11].

The trials that have EPA levels above 500 mg per day (Figure 1) also have DHA levels above 500 mg per day, except for the AREDS2 trial, where the dose of EPA (650 mg) was greater than 500 mg per day and greater than the dose of DHA (375 mg). As this trial had *n* significant results, it suggests that a dose of at least 500 mg of EPA is not as effective as a dose of at least 500 mg of DHA. To date, there are no clinical trials that have assessed EPA alone versus DHA alone on cardiac mortality. However it has been reported that low DHA levels are associated with increased all-cause mortality [36] and increased plasma DHA, but EPA is not associated with decreased progression of atherosclerosis in women with coronary artery disease [37].

### 3.2. Randomised Controlled Trials with Blood n-3 Assessments and Comparison to Omega-3 Index

The omega-3 index has been postulated as a risk factor for cardiovascular disease and it is expressed as EPA plus DHA as a percent of total erythrocyte fatty acids [38].

**Figure 2:**
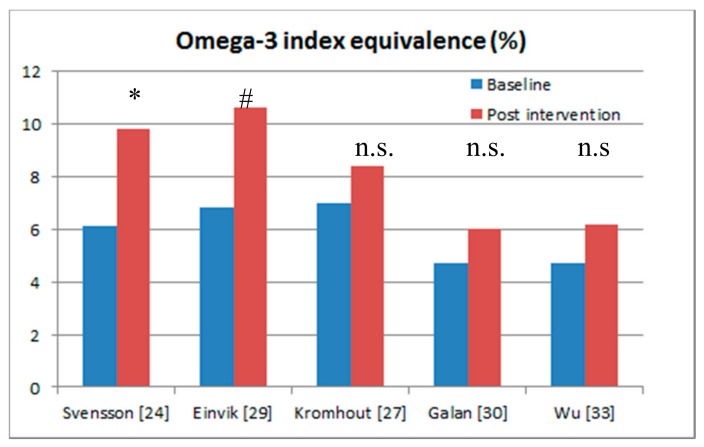
Randomised controlled trials of the effects of omega-3 supplementation that included blood sampling at baseline and post-intervention. * Significant effects (*p* < 0.05); ^#^ only 140 per group showing a trend towards a significant effect (*p* = 0.063); n.s: no significant effect.

Figure 2 and Table 1 show the five randomised controlled trials that included blood assessments of omega-3s in serum phospholipid fatty acids, serum fatty acids, plasma cholesteryl ester fatty acids, and plasma fatty acids, but none of these studies measured the omega-3 index in full blood [38]. For ease of comparison across studies, Stark et al. [39] have derived calculations to convert measurements of fatty acids in other blood fractions into the omega-3 index, measured in full blood, but these calculations have yet to be validated. Nevertheless, for ease of comparison the five studies with blood sampling have been converted to the omega-3 index equivalence (Table 1).

Svensson et al. (2006) [24] showed a 70% reduction in myocardial infarction and 60% reduction in major coronary events after omega-3 supplementation in people with chronic haemodialysis. The calculated omega-3 index [39] increased from 6% to 9.8%, which is considered cardioprotective [37]. In the study by Einvik et al. [29] there was a trend (*p* = 0.063) towards a 41% reduction in all-cause mortality and similar to Svensson et al. [24], the omega-3 index of 10.6% at the end of the trial was considered as cardio-protective [38].

The studies by Galan et al. [30] and Wu et al. [33] showed no significant cardiovascular benefits after omega-3 supplementation, but these studies failed to reach omega-3 fatty acid status levels that are associated with cardioprotection (i.e., between 8% and 11%) [38].

The omega-3 fatty acid status of 8.4% in the Alpha Omega Trial [27] did reach levels of cardioprotection [38], however the baseline omega-3 fatty acid status levels of 7% were relatively high and therefore an increment of only 1.4% (i.e., from 7% to 8.4%) is not high enough to see any benefit for CVD.

### 3.3. Importance of the Assessment of Omega-3 Fatty Acid Status Pre- and Post-Supplementation

Numerous factors, including supplementation time-span, polymorphisms in the genes involved in *n*-3 LCPUFA metabolism, dietary intake, and lifestyle habits can all influence omega-3 status, but this is not the focus of this manuscript. However, the importance of assessment of *n*-3 LCPUFA status is highlighted.

The study by Farquharson et al. [40] is an example of the importance of the assessment of omega-3 status pre- and post-supplementation. This short-term randomised placebo-controlled intervention trial assessed the effect of fish oil on atrial fibrillation after cardiac surgery that measured the omega-3 fatty acid status at baseline and post-intervention. The subjects ingested 15 mL of oil per day containing 2.7 g of EPA and 1.9 g DHA per day for 3 weeks prior to surgery and 6 days post-surgery. The mean omega-3 fatty acid status was not significantly different at baseline (5.96% control vs. 5.91% in fish oil groups) and only the fish oil group increased their omega-3 fatty acid status to 8.80% at surgery [40]. There was a trend towards an unadjusted hazards ratio for time to first episode of atrial fibrillation (AF) associated with fish oil of 0.66 (95% Confidence Interval (CI) 0.43–1.01, *p* = 0.06). The length of stay in hospital was significantly lower in the fish oil group (67 h) versus the control group (95 h) with an adjusted hazard ratio of means 0.71 (95% CI 0.56–0.90, *p* = 0.006) [40]. In this study, the researchers made every effort to exclude potential study participants from the trial due to high intakes of omega-3 (i.e., consumption of at least one fish meal per week or taking fish oil supplements), to ensure that the included study participants had low omega-3 status at the beginning of the trial. Even though there were some significant results obtained from fish oil supplementation, the authors pointed out that there was still considerable overlap of the omega-3 status between the placebo and fish oil group, with this overlap ranging from 5.5–9.0%, i.e., the upper 50% of the control group overlapped with the lower 50% of the fish oil group, with this overlap ranging from 5.5 to 9.0% [41]. Furthermore, the study participants that were excluded from the trial due to high omega-3 intakes had omega-3 levels indistinguishable from the fish oil group, and the omega-3 fatty acid status ranged from 5.5% to 13% in both groups (see Figure 2, [41]). Therefore, researchers assessing the effect of omega-3 supplementation must exclude potential study participants with high intakes of fish and those taking fish oil supplements. Furthermore, given the aforementioned considerable overlap in omega-3 status, rather than excluding potential study participants solely due to high dietary consumption of *n*-3 LCPUFA, it is pertinent that the blood omega-3 status be measured and people with high blood omega-3 levels are excluded from the trial. Therefore, clinical trials assessing the efficacy of *n*-3 LCPUFA supplementation should assess *n*-3 LCPUFA levels pre- and post-supplementation and exclude people with high levels of omega-3 index to prevent obtaining a potential ceiling effect.

### 3.4. Evidence from Epidemiological Studies

Bell et al. [42] showed that the highest quartile of *n*-3 LCPUFA intake was associated with a small decreased risk of total mortality and a non-significant reduction of CVD mortality. One reason for the minimal benefit could be that the highest quartile of total *n*-3 LCPUFA intake was only 323 mg per day or higher. Even though benefits are seen in quartile 4 of *n*-3 LCPUFA intakes (and not with quartiles 2 and 3), large risk reductions are not shown because the total intakes were so low at 323 mg total *n*-3 LCPUFA per day, and therefore DHA intake would be even lower than 323 mg per day. This highlights the importance of DHA dose.

Del Gobbo et al. [43] pooled 19 cohort studies and investigated the *n*-3 biomarkers and coronary heart disease, concluding that biomarker concentrations of *n*-3 PUFA are associated with a lower incidence of fatal coronary heart disease. The biomarkers used in these 19 cohort studies included plasma phospholipids, erythrocyte phospholipids, total plasma, cholesterol esters, and adipose tissue (see online supplement to [43]). There were two cohort studies from the 19 that measured erythrocyte phospholipid fatty acids in a manner comparable to the omega-3 index. The median quintile (Q) comparison for Q1 versus Q5 for erythrocyte EPA showed values of 0.28% and 1.08% respectively; the median quintile comparison for Q1 versus Q5 for erythrocyte DHA showed values of 1.99% and 5.15%, respectively, and the quintile comparison for Q1 versus Q5 for the omega-3 index showed values of 2.27% and 6.22%, respectively. The highest quintile (Q5) had an omega-3 index of only 6.22%, which is lower than the 8% stipulated for cardioprotection [38]. The highest Q5 of the other biomarkers was also quite low, indicating that populations are low in their *n*-3 status.

Both the Bell et al. [42] and the Del Gobbo [43] studies highlight that the actual intakes of *n*-3 LCPUFA and biomarkers in the highest quartile and quintile, respectively, demonstrated beneficial effects of *n*-3 LCPUFA, even though the intakes and omega-3 index were low. This suggests that the dose and hence the *n*-3 status is important. The epidemiological studies support the RCTs in that perhaps a minimum dose of 500 mg of DHA is required for cardioprotection.

### 3.5. Duration of Interventions

More recently, the GISSI-Heart Failure (HF) study (840 mg per day; 460 mg EPA and 380 mg DHA) reported the omega-3 fatty acid status increased from 4.8 ± 1.7% to 6.7 ± 1.9% after 3 months of supplementation, with only 23% of patients reaching the target cardioprotection level of ≥8% [44]. This suggests that perhaps 3 months may not be long enough as the half-life of erythrocytes is 120 days. This study definitely also highlights the importance of the intervention duration and we want to make readers aware of its importance as well. Study duration is not the focus of the current study, but deserves attention in future review studies.

Moreover, this study highlights the necessity to analyse on a per-protocol basis rather than relying on only intention-to-treat (ITT) analysis.

### 3.6. Evidence for the Beneficial Effect of High Dose n-3 LCPUFA Post-Myocardial Infarction

Heydari et al. [45] showed that high-dose supplementation (1860 mg EPA and 1500 mg DHA per day) for 6 months in patients who have suffered a myocardial infarction was associated with reduced adverse left ventricular remodelling, non-infarct myocardial fibrosis, and serum markers of systemic inflammation beyond current guideline-based standards of care. This high dose supplementation for a 6-month duration increased the patients’ omega-3 index from 5.5% (at the time of myocardial infarction) to 10%. This study supports the importance of *n*-3 LCPUFA for cardiovascular health and it demonstrates that the dose required is much higher to aid in recovery after a myocardial infarction. This study also demonstrates the importance of assessing blood levels to confirm excellent compliance and that *n*-3 LCPUFAs do have a role in repair (e.g., left ventricular remodelling) after a myocardial infarction. A much higher dose is required post-myocardial infarction, whereas it appears that 500 mg DHA daily is required to prevent death from cardiovascular disease.

## 4. Summary

In summary, the RCTs that included a sufficient dose of DHA (at least 500 mg per day) showed reductions in cardiovascular mortality. The ITT analysis is the gold standard for analysing RCTs, which were originally designed to study the effects of drugs, where the placebo group equated to no drug at all and the test group was exposed to the drug. However, this is not that clear-cut when conducting *n*-3 LCPUFA supplementation trials, as they are conducted in the context of both the placebo group and the active group being exposed to *n*-3 LCPUFA previously by nutritional intake, and hence there is no true placebo group (i.e., group with no drug exposure) [41]. Therefore, it is advisable to also analyse the data on a per-protocol basis. Furthermore, the RCTs that did take blood samples and those studies that reached an omega-3 index equivalence of 8% or greater showed cardioprotection. Therefore, when assessing the effect of omega-3 supplementation to determine a health benefit, it is pertinent to measure the omega-3 fatty acid status in the blood at baseline and post intervention and it is advisable to exclude people with very high baseline omega-3 fatty acid status from such research trials to achieve adequate power and to prevent a potential ceiling effect.

Albeit based on one clinical trial, it appears that much higher doses (1860 mg EPA and 1500 mg DHA per day) for a 6-month duration are required to improve recovery after myocardial infarction, suggesting that much higher doses are required to ‘treat’ rather than to ‘prevent’ cardiovascular disease.

## 5. Conclusions

For both clinicians and researchers, the message is that a sufficient dose of DHA of at least 500 mg per day should be provided to reduce cardiovascular mortality; that blood samples should be taken pre- and post-supplementation of *n*-3 LCPUFA; and the background fatty acid status should be measured to prevent a potential ceiling effect of *n*-3 LCPUFA supplementation. Furthermore, researchers should be aware that supplementation trials must be analysed on a per-protocol basis in addition to ITT analysis in order to show an efficacy that can be attributed to *n*-3 LCPUFAs.

## Figures and Tables

**Table 1:** Randomised controlled trials (RCTs) of the effect of omega-3 supplementation that included blood sampling at baseline and post-intervention.

Reference	Blood Sample Type	Equation and Pearson’s Correlation [39]	Omega-3 Index Equivalence at Baseline	Omega-3 Index Equivalence Post-Intervention	Outcomes
Svensson et al. 2006 [24]	Serum phospholipid fatty acids	Y = 0.93x + 0.55 *R* = 0.94	6.1%	9.8%	Myocardial infarction 3.9% versus 12.6% (*p* = 0.036) Major coronary events 6.8% versus 16.5% (*p* = 0.043) Note the large increase in omega-3 fatty acid status (38%) achieved Omega-3 fatty acid status is in the cardio-protective zone.
Einvik et al. 2010 [29]	Serum fatty acids	Y = 0.94x + 1.17 *R* = 0.74	6.8%	10.6%	All-cause mortality 5% versus 8.5% (*p* = 0.063) Note the large increase in omega-3 fatty acid status (36%) achieved Omega-3 fatty acid status is in the cardio-protective zone.
Kromhout et al. 2010 [27]	Plasma cholesteryl esters fatty acids	Y = 1.59x + 2.05 *R* = 0.85	7.0%	8.4%	No significant outcomes. Note the small increase in omega-3 fatty acid status (17%) achieved.
Galan et al. 2010 [30]	Plasma fatty acids	Y = 0.94x + 1.17 *R* = 0.74	4.7%	6.0%	No significant outcomes. Note the small increase in omega-3 fatty acid status (22%) achieved. Omega-3 fatty acid status of 6% is not in the cardio-protective zone
Wu et al. 2013 [33]	Serum phospholipid fatty acids	Y = 0.93x + 0.55 *R* = 0.94	4.7%	6.2%	No significant outcomes. Note the small increase in omega-3 fatty acid status (24%) achieved. Omega-3 fatty acid status of 6% is not in the cardio-protective zone

## Supplementary Materials

The following are available online at www.mdpi.com/2072-6643/9/12/1305/s1. Table S1: Randomised controlled trials of *n*-3 LCPUFA and CVD mortality (including CHD, stroke, heart failure, sudden cardiac death).
