# Supplementary material for: Effects of Omega-3 Long Chain Polyunsaturated Fatty Acid Supplementation on Cardiovascular Mortality: The Importance of the Dose of DHA
Source: Nutrients. 2017 Nov 30;9(12):1305. doi: 10.3390/nu9121305 (PMC5748755; doi:10.3390/nu9121305)
Supplement: Supplementary file 1 [file nutrients-09-01305-s001.zip › nutrients-231302-supplementary.pdf]

nutrients

MDPI

Table S1. Randomised Controlled Trials of n-3 LCPUFA and CVD mortality (includes CHD, Stroke, Heart failure, Sudden Cardiac death)

Ref	n-3 Dose & placebo	duration	Study pop & no. & year of study	Anti-platelet	ACE Inhibitors	B-blockers	Chol drugs?	Diet?	Blood n-3s?	Outcome measures	Comments
GISSI-P Lancet 1999 [1]	280mg EPA 560mg DHA per day. Placebo - nothing	3.5 years	11,324 (=2,831 per group) males with previous MI Oct 93-Sept 95	92% B 88% 6 83% 42 mths	47% B 41% 6 40% 42 mths	44% B 41% 6 39% 42 mths	5% B 29% 6 45% 42 mths	≥ 1 fish serve/wk = 73% B =87% 6 & 42 mths	No	<u>Primary</u><br>Death<br>(actual events 12.3% vs 14.6%)<br>Non-fatal MI<br>Stroke<br><u>Secondary analysis</u><br>All fatal events<br>CVD death<br>Cardiac<br>Coronary<br>Sudden death	Death 15% ↓<br>CVD death 20% ↓<br>↓ 20%<br>↓ 30%<br>↓ 35%<br>↓ 35%<br>↓ 40%
Svensson *et al.* 2006 OPACH study [2]	Omacor - 2 465mg EPA 375mg DHA Therefore: 930mg EPA 750mg DHA Placebo Olive Oil	558 days median follow-up (1.5 years)	206 (n=103 per group)<br>Chronic haemodialysis patients Nov 2002-May 2003	70%	25%	53%	20%	No	Yes	<u>Primary:</u><br>All-cause mortality<br>Total CV events<br>(actual events 60% vs 57%)<br><u>Secondary:</u><br>MI (actual events 3.9% vs 12.6%)	n.s.<br>↓ 70%<br>↓ 60%

										Major coronary events (actual events 6.8% vs 16.5%)	
JELIS Lancet 2007 [3]	1800mg EPA per day & statin Or statin alone (=placebo)	5 years	18,645 (=9,323 per group) (69% women) Mean age 61 years TC≥6.5mmol/L 1996-1999	14% B	25%	19% B	98%	No	No	Primary<br>Any major coronary event (actual events 2.8% vs 3.5%)<br>Unstable angina<br>Non-fatal coronary events	2.8% EPA<br>3.5% controls<br>= ↓ 19%<br>= ↓ 24%<br>= ↓ 19%<br>Secondary prevn
GISSI-HF Lancet 2008 [4]	280mg EPA 560mg DHA per day. Matching placebo.	5 years	6,975 (=3,488 per group) (22% women) 6 Aug 2002- 28 Feb 2005	10%	77%	65%	23%	No	No	Primary<br>Time to death (actual events 27% vs 29%)<br>Admission to hospital<br>Secondary<br>CV mortality admission for any reason, sudden cardiac death, admission for cardio vascular reasons, admission for HF, MI, and stroke	= ↓ 9%<br>= ↓ 8%<br>= ↓ 10%<br>= ↓ 7%

Einvik *et al.*<br>Euro J<br>Cardiovasc<br>Prev<br>Rehabil<br>2010<br>[5]	4 groups:<br>Placebo no diet<br>Placebo & diet<br>n-3 no diet<br>n-3 & diet<br><br>1176mg EPA<br>840mg DHA<br>Placebo corn oil	3 years	563 men<br>(=approx. 140 per group)<br>64-76 years<br>High cholesterol<br>>6.45mmol/l	Not reported	Not reported	Not reported	Not reported	No	Yes	<u>Primary:</u><br>Carotid IMT<br>All-cause mortality<br>(actual event rate 5% vs 8.5%, p=0.06)<br>CV events<br>(actual event rate 11% vs 12.8%, p=0.6)	The DOIT was designed and powered to investigate the effect of the intervention strategies on progression of atherosclerosis and thus, with insufficient power to detect changes in mortality.<br>Placebo group were significantly younger!!<br>Atherosclerosis largest risk factor is age!!!<br>The authors recognise the small sample size!!<br>Also change in EPA 2.7fold<br>Change in DHA 1.4 fold
Kromhout<br>2010 Alpha<br>Omega<br>Trial<br>[6]	Margarine 18.8g plus<br>1. 376mg EPA and DHA 3:2<br>= 226mg EPA & 150mg DHA<br>2. 1.9g ALA	40 months	4,873 (=1,200 per group)<br>Aged 60-80<br>78% men who had MI<br>April 2002-Dec 2006	97%	90%	90%	86%	No	Yes	<u>Primary:</u><br>Rate of CV event (actual events 4.6% vs 4.57%)<br>MI<br>Cardiac arrest<br>Stroke<br>Cardiac interventions:	The power was only 35% due to the limited number of cases that

	3. 1.9g ALA<br>226mg EPA<br>150mg DHA 4.<br>Placebo									PCI<br>CABG<br>Debifr<br><br>Non fatal CV events	occurred in the Alpha Omega Trial.<br>**Because it is inadequately powered you cannot use it is a null finding**
Rauch Circulation 2010<br>Omega trial<br>[7]	Purified omega-3<br>(460mg EPA<br>380mg DHA)<br>in addition to current guidelines for MI<br>Placebo olive oil	1 year	3,851<br>(=1,926 per group)<br>Aged 60<br>All had MI<br>25.6% female<br>Oct 2003-<br>June 2007		83%	94%	94%	Start:<br>No 4%<br>Occ 67%<br>Sev 28%<br>Daily .3%<br>End:<br>No 3%<br>Occ 51%<br>Sev 45%<br>Daily .8%	No	<u>Primary:</u><br>Sudden cardiac death (actual events 1.5% vs 1.5%)<br><u>Secondary:</u><br>Total mortality<br>Nonfatal clinical events	They did not reach statistical power!!<br>The presented data therefore do<br>not allow a final answer on the potential benefit of the additional application of highly purified omega-3 fatty acids for secondary prevention after acute myocardial infarction.<br>**Because it is inadequately powered you cannot use it as a null finding**
Galan BMJ<br>2010<br>[8]	B vits & n-3s<br>(400mg EPA<br>200mg DHA)	4.7 year	2,501<br>(=approx. 620 per group)	93%	53%	68%	86%		Yes	<u>Primary:</u><br>CV event (actual event rate 6.3%)	Not significant

	n-3s<br>(400mg EPA<br>200mg DHA)<br>B vits<br>Placebo		Men & women aged 45-80 who had acute coronary or ischaemic event in past 12 mths<br>(MI or acute coronary syndrome)							Bloods (% of tot FA)<br>EPA 1.15 to 2.1%<br>DHA 2.5 to 3.1%<br>EPA & DHA<br>3.7 to 5.2%	↑ 0.95% EPA<br>↑ 0.6% DHA<br>↑ 1.5% EPA & DHA
Origin<br>LVEF<br>2011<br>[9]	465mg EPA<br>375mg DHA<br><br>3 groups:<br>4g n-3/day<br>1g n-3 & 3g placebo/day<br>4g placebo/ day	3 mths	43<br>(pilot study)<br>Severe non-ischemic HF		75-93%<br>ACE or ARB<br>100%	100%		No	No	<u>Primary:</u><br>LVEF<br>FMD<br>IL-6<br>TNF-a<br>Exercise peak O2 consumption	Dose response<br>LVEF<br>FMD<br>IL-6<br>TNF-a going in right direction but n.s.<br>Exercise n.s.<br>1g dose = 375mg DHA<br>4g dose = 1500mg DHA
Origin<br>NEJM<br>2012<br>[10]	465mg EPA<br>375mg DHA<br>Placebo olive oil	6.2 years	12,536<br>(approx. 6,300 per group)<br>Patients with dysglycemia	70%	69%	53%	54%	No, except n-3 supplements was discouraged<br>FFQ B, 2 yr and end	No	<u>Primary:</u><br>Death for CV causes (actual event rate 9.1 vs 9.3%)<br><u>Secondary:</u><br>Death from arrhythmia (actual event rates 0.78 vs 0.70)	Event rates are too low to draw any

											meaningful conclusions
OPERA trial<br>Wu *et al.*<br>J Am Heart Assoc<br>2013<br>[11]	465mg EPA<br>375mg DHA<br>(8-10g total)<br><br>Placebo olive oil	No. of FO loading days<br>1=3<br>2=108<br>3=51<br>4=32<br>5=73<br>Plateau 4-5 days	564<br>aged 63<br>72% male<br>Aug 2010-<br>June 2012	61%	35%	53%	56%	No	Yes	Primary:<br>PoAF	DHA is better for AF than EPA.<br><br>An ↑in DHA from 3.15 to 3.84% is too low to see an effect on AF.
Risk and Prevention Study<br>NEJM 2013<br>[12]	465mg EPA<br>375mg DHA<br><br>Placebo olive oil	5 years	12,513<br>(=6,244 per group)	41%	45%	20%	41%	No	No	Primary:<br>Rate of death<br>(actual event rate 11.7% vs 11.9%, p=0.64)<br><br>Nonfatal MI<br>Nonfatal stroke	However, after a blinded assessment at 1 year showed an event rate that was lower than expected, the primary efficacy end point was revised as the composite of time to death from cardiovascular causes or hospital admission for cardiovascular

											causes. There were significantly fewer admissions for heart failure among patients who received n−3 fatty acids than among those who received placebo (96 patients [1.5%] vs. 142 patients [2.3%], P = 0.002) (Table S5 in the Supplementary Appendix). Women: a significantly lower rate of events among those who received n−3 fatty acids than among those who received placebo (hazard ratio, 0.82; 95% CI, 0.67 to 0.99; P = 0.04) (Fig. 2).

											No dietary intake assessments and no bloods. Possible overlap of study groups in terms of omega-3 status.<br>No compliance check!
AREDS2 [13]	650mg EPA<br>350mg DHA<br>10mg lutein<br>2mg zeaxanthin<br>placebo	4.8 years	Cardiovascular Outcome Study was an ancillary study to the Age-Related Eye Disease Study 2 (AREDS2)<br>N=4,203<br>Aged 50-85 years<br>Intermediate or advanced ARMD in one eye	Not reported	Not reported	Not reported	Not reported	No	Not reported	Cardiovascular disease mortality (sudden death; death due to MI, heart failure, or stroke);<br>CVD morbidity (MI; stroke; unstable angina; coronary and carotid revascularization; hospitalized CHF; resuscitated cardiac arrest).	Only 9% event rates in n-3 and placebo groups<br>Unadjusted HR (95% CI)<br>0.95 (0.78-1.17).<br>Authors admit that the study was powered to detect a change in macular degeneration and underpowered for CVD outcomes.

1. GISSI Prevezione Investigators. Dietary supplementation with n-3 polyunsaturated fatty acids and vitamin e after myocardial infarction: Results of the gissi-prevenzione trial. *The Lancet* **1999**, *354*, 447-455.
2. Svensson, M.; Schmidt, E.B.; Jorgensen, K.A.; Christensen, J.H.; Group, O.S. N-3 fatty acids as secondary prevention against cardiovascular events in patients who undergo chronic hemodialysis: A randomized, placebo-controlled intervention trial. *Clin J Am Soc Nephrol* **2006**, *1*, 780-786.
3. Yokoyama, M.; Origasa, H.; Matsuzaki, M.; Matsuzawa, Y.; Saito, Y.; Ishikawa, Y.; Oikawa, S.; Sasaki, J.; Hishida, H.; Itakura, H., *et al.* Effects of eicosapentaenoic acid on major coronary events in hypercholesterolaemic patients (jelis): A randomised open-label, blinded endpoint analysis. *The Lancet* **2007**, *369*, 1090-1098.
4. GISSI-HF Investigators. Effect of n-3 polyunsaturated fatty acids in patients with chronic heart failure (the gissi-hf trial): A randomised, double-blind, placebo-controlled trial. *The Lancet* **2008**, *372*, 1223-1230.
5. Einvik, G.; Klemsdal, T.O.; Sandvik, L.; Hjerkinn, E.M. A randomized clinical trial on n-3 polyunsaturated fatty acids supplementation and all-cause mortality in elderly men at high cardiovascular risk. *Eur J Cardiovasc Prev Rehabil* **2010**, *17*, 588-592.
6. Kromhout, D.; Giltay, E.J.; Geleijnse, J.M. N-3 fatty acids and cardiovascular events after myocardial infarction. *N Engl J Med* **2010**, *363*, 2015-2026.
7. Rauch, B.; Schiele, R.; Schneider, S.; Diller, F.; Victor, N.; Gohlke, H.; Gottwik, M.; Steinbeck, G.; Del Castillo, U.; Sack, R., *et al.* Omega, a randomized, placebo-controlled trial to test the effect of highly purified omega-3 fatty acids on top of modern guideline-adjusted therapy after myocardial infarction. *Circulation* **2010**, *122*, 2152-2159.
8. Galan, P.; Kesse-Guyot, E.; Czernichow, S.; Briancon, S.; Blacher, J.; Hercberg, S.; Group, S.F.O.C. Effects of b vitamins and omega 3 fatty acids on cardiovascular diseases: A randomised placebo controlled trial. *Br Med J* **2010**, *341*, c6273.
9. Moertl, D.; Hammer, A.; Steiner, S.; Hutuleac, R.; Vonbank, K.; Berger, R. Dose-dependent effects of omega-3-polyunsaturated fatty acids on systolic left ventricular function, endothelial function, and markers of inflammation in chronic heart failure of nonischemic origin: A double-blind, placebo-controlled, 3-arm study. *Am Heart J* **2011**, *161*, 915 e911-919.
10. The Origin Trial Investigators. N-3 fatty acids and cardiovascular outcomes in patients with dysglycemia. *N Engl J Med* **2012**, *367*, 309-318.
11. Wu, J.H.; Marchioli, R.; Silletta, M.G.; Macchia, A.; Song, X.; Siscovick, D.S.; Harris, W.S.; Masson, S.; Latini, R.; Albert, C., *et al.* Plasma phospholipid omega-3 fatty acids and incidence of postoperative atrial fibrillation in the opera trial. *J Am Heart Assoc* **2013**, *2*, e000397.
12. The Risk and Prevention Study Collaborative Group. N-3 fatty acids in patients with multiple cardiovascular risk factors. *N Engl J Med* **2013**, *368*, 1800-1808.
13. Writing Group for the AREDS2 Research Group. Effect of long-chain omega-3 fatty acids and lutein + zeaxanthin supplements on cardiovascular outcomes: Results of the age-related eye disease study 2 (areds2) randomized clinical trial. *JAMA Intern Med* **2014**, *174*, 763-771.
